# Prevalence and associated factors of depressive symptoms among older adult diabetic patients in China: a nationally representative cross-sectional study

**DOI:** 10.3389/fpsyg.2025.1581603

**Published:** 2025-06-27

**Authors:** Huan Huang, Wu-xiao Wei, Tao Huang, Feng Wang, Hai-tao Zhang

**Affiliations:** ^1^People's Hospital of Liuzhi Special District, Liupanshui, China; ^2^Guangxi University of Science and Technology First Affiliated Hospital, Liuzhou, China; ^3^Liuzhou Municipal Liutie Central Hospital, Liuzhou, China

**Keywords:** older adults, diabetes, depressive symptoms, sociodemographic factors, chronic diseases, CHARLS

## Abstract

**Background:**

Diabetes and depressive symptoms exhibit a high comorbidity in the older adult population, and their combined effects significantly reduce patients’ quality of life. This study aims to investigate the prevalence of depressive symptoms among older adults with diabetes in China and identify key associated factors, providing evidence-based insights for the development of targeted intervention strategies.

**Methods:**

This study utilizes data from the 2015, 2018, and 2020 waves of the China Health and Retirement Longitudinal Study (CHARLS), including older adults aged 60 years and above who have been diagnosed with diabetes (*n* = 3,609). Depressive symptoms were assessed using the simplified version of the CES-D scale, a validated tool for measuring depressive symptoms, with a score of ≥10 indicating depressive symptoms. Univariate chi-square tests and logistic regression analysis were used to examine the factors associated with depressive symptoms, with statistical significance set at *p* < 0.05.

**Results:**

The overall prevalence of depressive symptoms among older adult diabetic patients in the sample was 38.6%. Univariate chi-square analysis revealed significant differences in variables including age (*χ*^2^ = 4.048, *p* = 0.044), gender (*χ*^2^ = 96.725, *p* < 0.001), educational level (*χ*^2^ = 110.545, *p* < 0.001), and sleep duration (*χ*^2^ = 161.070, *p* < 0.001) between the groups with and without depressive symptoms. Logistic regression analysis identified lower educational level (OR = 1.26, 95% CI: 1.03–1.55, *p* = 0.026), female sex (OR = 1.51, 95% CI: 1.22–1.88, *p* < 0.001), and shorter sleep duration (OR = 1.76, 95% CI: 1.41–2.18, *p* < 0.001) as independent risk factors for depressive symptoms. Furthermore, patients with comorbidities had a significantly increased risk of depressive symptoms (OR = 1.35, 95% CI: 1.06–1.72, *p* = 0.015).

**Conclusion:**

The high prevalence of depressive symptoms among older adult diabetic patients is significantly associated with sociodemographic, lifestyle, and health-related factors. Personalized psychological interventions should be prioritized for high-risk groups, including individuals with low education levels, women, those experiencing insufficient sleep, and those with multiple comorbidities, in order to enhance their quality of life and reduce social burdens.

## Introduction

Depressive symptoms are a highly prevalent mental disorder that profoundly impact the physical and mental health of older adults worldwide (Alexopoulos, 2005; Fiske et al., 2009; Hu et al., 2022). With the accelerating growth of the elderly population, the prevalence of depressive symptoms among older adults continues to rise, particularly among those with diabetes, where the situation is even more pronounced (Li et al., 2024; Zhang C. et al., 2023). Evidence suggests that the risk of developing depressive symptoms in diabetic patients is significantly higher than in the general population. This comorbidity not only reduces the quality of life for patients but also increases the burden on families and complicates disease management and clinical interventions (Zhang H. et al., 2023; Liu et al., 2020). Among elderly diabetic patients, the prevalence of depressive symptoms is notably higher, with approximately 30% exhibiting depressive symptoms and 12–18% meeting the diagnostic criteria for major depressive disorder, a proportion substantially higher than that in the non-diabetic population (Li et al., 2024; Soleimani et al., 2021). This high comorbidity rate presents a significant challenge to public health policymakers and healthcare providers.

The relationship between depressive symptoms and diabetes is bidirectional. On one hand, the persistent hyperglycemic burden and chronic complications in diabetic patients may trigger or exacerbate depressive symptoms (Lustman et al., 2000). On the other hand, depressive symptoms themselves negatively impact diabetes management, as individuals with depressive symptoms often neglect self-care and medication adherence, leading to fluctuations in blood glucose levels and an increased risk of complications (Varela-Moreno et al., 2022). Furthermore, depressive symptoms are a common comorbidity in elderly diabetic patients and often coexists with other chronic diseases. Studies have shown that depressive symptoms are not only prevalent in diabetes but are also closely associated with hypertension, heart disease, and stroke (Mezuk et al., 2008; Roy and Lloyd, 2012; Liu et al., 2022). The presence of these comorbidities complicates patient management, as they increase both the physical and psychological burdens.

In the United States, a significant bidirectional relationship exists between diabetes and depressive symptoms (Golden et al., 2008). Research indicates that diabetic patients are twice as likely to develop depressive symptoms as the general population, with a particularly higher incidence of depressive symptoms among those with type 2 diabetes. The need for long-term management of blood glucose, diet, and lifestyle in diabetic patients may contribute to psychological stress and emotional distress, increasing the risk of depressive symptoms. Meanwhile, depressive symptoms interfere with diabetes self-management, leading to neglect of health management, reduced physical activity, and irregular eating habits, thereby affecting blood glucose control (Anderson et al., 2001; Golden et al., 2008). In a five-year longitudinal epidemiological study conducted in Spain, depressive symptoms were considered an independent risk factor for the development of diabetes, and the study demonstrated that depressive symptoms significantly increased the risk of developing diabetes, especially in cases of non-severe depression, persistent depression, and untreated depressive symptoms (Campayo et al., 2010). In China, research on the intersection of diabetes and depressive symptoms in the elderly is still emerging. Among individuals with type 2 diabetes in China, 25.9% experience varying degrees of depressive symptoms, with significant heterogeneity across different studies. Factors such as female gender, age ≥ 60 years, low education (especially at or below elementary school level), diabetes duration ≥ 10 years, presence of complications, insulin use, and living alone are significantly associated with the occurrence of depressive symptoms (Liu et al., 2022; Ju et al., 2022).

Therefore, utilizing large-scale data from the China Health and Retirement Longitudinal Study (CHARLS), this article analyzes data from 2015, 2018, and 2020 to reveal the prevalence of depressive symptoms among elderly diabetic patients. It provides an in-depth exploration of the depressive symptoms and their influencing factors in elderly diabetic patients in China, offering important baseline data for related fields. The findings of this study will provide empirical support for the development of mental health interventions and contribute to the advancement of tailored public health policies for elderly diabetic patients.

## Methods

### Study design and data source

This study investigates the prevalence of depressive symptoms and their major influencing factors among Older Adults diabetic patients in China, using data from the CHARLS. CHARLS is a nationally representative longitudinal survey designed to collect high-quality microdata on individuals aged 45 years and older, focusing on issues related to population aging (Zhao et al., 2014). The baseline survey in 2011 employed a multistage probability sampling method with probability proportional to size (PPS), covering 450 villages, 150 counties, and 28 provinces, with over 17,000 individuals from approximately 10,000 households. Follow-up surveys were conducted every two to three years, with four waves released to date: 2011 (Wave 1), 2013 (Wave 2), 2015 (Wave 3), and 2018 (Wave 4). The data are publicly available at the CHARLS website^1^.

**Figure 1:**
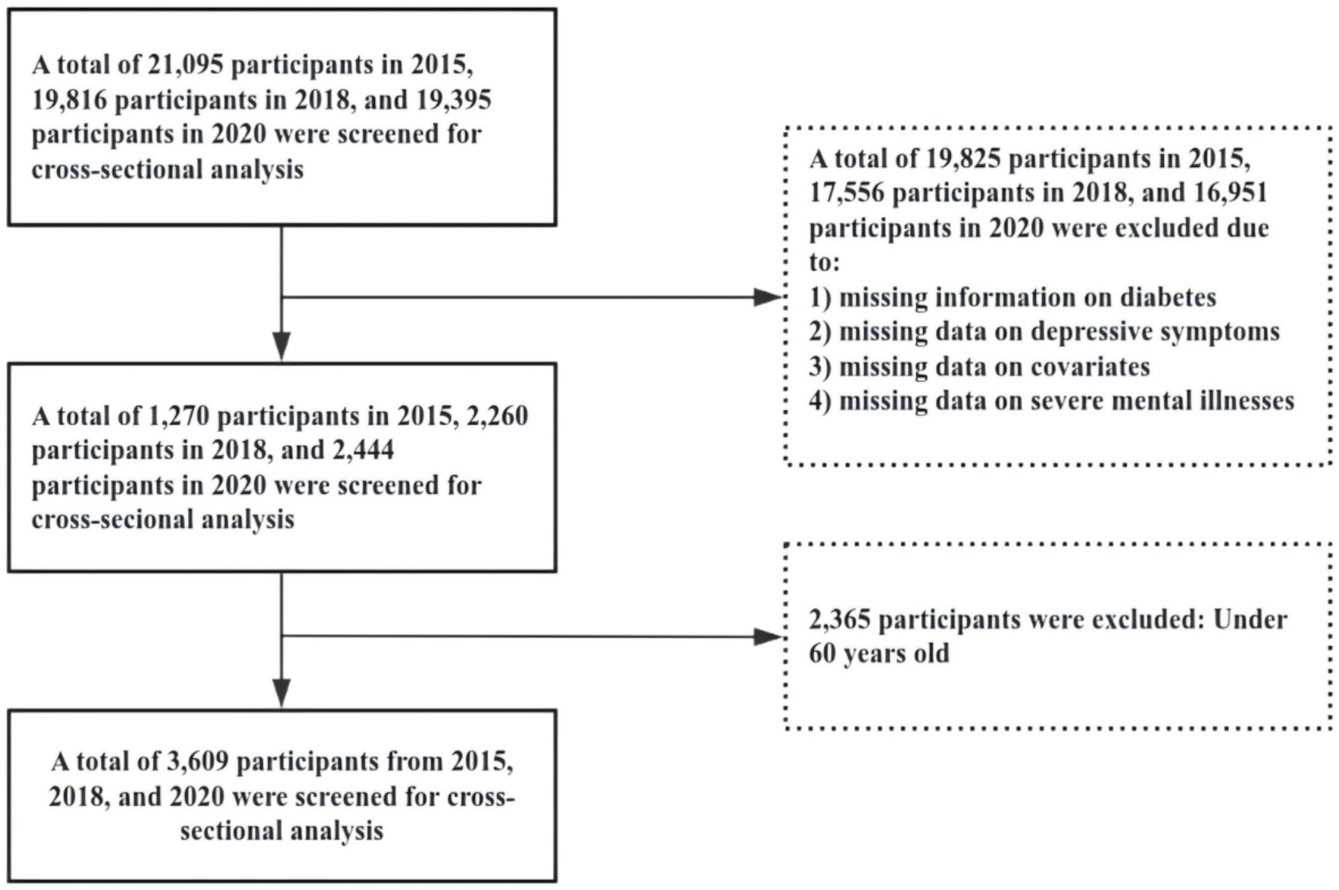
Shows a detailed flowchart of the sample selection process.

This study utilized data from the CHARLS for the years 2015, 2018, and 2020. The inclusion criteria were as follows: (1) age ≥ 60 years; (2) affirmative response to the question: “Have you ever been diagnosed with diabetes or elevated blood glucose (including impaired glucose tolerance and impaired fasting glucose) by a doctor?”; (3) provision of valid responses to the Center for Epidemiologic Studies Depression (CES-D) Scale. Data preprocessing excluded samples with missing information on diabetes, depression, or covariates. To ensure data quality, individuals with severe mental illnesses were also excluded. Figure 1 illustrates the detailed sample selection process.

### Assessment of depressive symptoms

The CHARLS study utilized the abbreviated version of the CES-D (Boey, 1999; Mohebbi et al., 2018), originally developed by Radloff. This scale has demonstrated high reliability and validity in previous studies and is widely used in older adult populations (Dye et al., 1995). The CES-D comprises 10 items (Song et al., 2023) rated on a four-point Likert scale: “3 = Always,” “2 = Often,” “1 = Sometimes or Rarely,” and “0 = Never,” with items 5 through 8 reverse scored. The total score ranges from 0 to 30. Based on prior research in older adult populations, a threshold score of ≥10 was adopted in this study to classify the presence of depressive symptoms.

### Covariates

Data collected from all study participants included the following variables: age, education level, gender, marital status, region, residence area, smoking, drinking, sleep duration, and the presence of chronic conditions, such as hypertension, dyslipidemia, cancer, pulmonary disease, liver disease, heart disease, stroke, kidney disease, and asthma.

### Statistical analysis

Descriptive statistics were used to analyze the distribution of the sample’s basic characteristics. Chi-square tests were applied to assess the significance of differences between the dependent variable (depressive symptoms) and explanatory variables (e.g., age, educational level, gender). For multivariate analysis, logistic regression models were employed to examine the effects of various factors on depressive symptoms, with results reported as odds ratios (OR) and 95% confidence intervals (CI). Sensitivity analyses were conducted to ensure the robustness of the model by reclassifying depressive symptoms using alternative CES-D score thresholds (e.g., scores of 8 and 12) and constructing additional regression models to verify the consistency of results. All statistical analyses were performed using Stata 16.0 software, with statistical significance set at *p* < 0.05. The coding scheme for the independent variables is provided in Table 1.

**Table 1:** Assignment of independent variables.

Variable type	Variable	Assignment
Dependent variable	Depressive	Assessed using the depression scale in CHARLS (CES-D 10 scale). If the CES-D score is greater than 10, it is assigned a value of 1; otherwise, it is assigned a value of 0.
Independent variable	Age	Ages 61 to 80 = 1, ages 81 and above = 2
	Education level	Primary school and below = 1, middle school = 2, high school and above = 3
	Gender	Male = 1, Female = 0
	Marital status	Married = 1, Divorced/Widowed/Separated/Single = 0
	Region	Urban areas = 1, Rural areas = 0
	Residence area	Eastern region = 1, Central region = 2, Western region = 3
	Smoking	Yes = 1, No = 0
	Drinking	Yes = 1, No = 0
	Sleep duration	Short sleep duration (<6 h/day) = 1, Normal sleep duration (6–8 h/day) = 2, Long sleep duration (>8 h/day) = 3
	Hypertension	Yes = 1, No = 0
	Dyslipidemia	Yes = 1, No = 0
	Cancer	Yes = 1, No = 0
	Pulmonary disease	Yes = 1, No = 0
	Liver disease	Yes = 1, No = 0
	Heart disease	Yes = 1, No = 0
	Stroke	Yes = 1, No = 0
	Kidney disease	Yes = 1, No = 0
	Asthma	Yes = 1, No = 0

## Results

### Descriptive analysis

Table 2 presents the baseline characteristics of older adult diabetic patients with depressive symptoms. Among the participants, 95.15% were aged between 61 and 80 years, while 4.85% were aged 81 years or older. Most participants (70.38%) had an educational level of elementary school or below, 16.68% had attended middle school, and only 12.94% had completed high school or higher education. Women accounted for 56% of the sample, while men made up 44%. In terms of marital status, 78.69% were married, while 21.31% were divorced, widowed, separated, or single. Rural and urban areas was nearly evenly distributed, with 50.12% living in rural areas and 49.88% in urban areas. Regionally, 37.05% of participants resided in western China, 38.54% in central China, and 24.41% in eastern China. Regarding health-related behaviors, 40.51% of participants were smokers, and 26.82% reported alcohol consumption. Sleep duration was categorized into three groups: 32.06% had short sleep (<6 h/day), 44.36% had normal sleep (6–8 h/day), and 23.58% had long sleep (>8 h/day). Chronic conditions were highly prevalent, with 66.08% having hypertension and 51.07% having dyslipidemia. Other common chronic diseases included Pulmonary disease (18.43%), heart disease (37.38%), and kidney disease (17.18%).

**Table 2:** Basic characteristics analysis.

Category	Subcategory	Total (*n* = 3,609)	
		Sample size	Proportion (%)
Age	61–80 years	3,434	95.15
	≥81 years	175	4.85
Education level	Primary school and below	2,540	70.38
	Middle school	602	16.68
	High school and above	467	12.94
Gender	Female	2,021	56
	Male	1,588	44
Marital Status	Divorced/Widowed/Separated/Single	769	21.31
	Married	2,840	78.69
Region	Rural areas	1,809	50.12
	Urban areas	1,800	49.88
Residence area	Eastern region	1,337	37.05
	Central region	1,391	38.54
	Western region	881	24.41
Smoking	No	2,147	59.49
	Yes	1,462	40.51
Drinking	No	2,641	73.18
	Yes	968	26.82
Sleep duration	Short sleep duration	1,157	32.06
	Normal sleep duration	1,601	44.36
	Long sleep duration	851	23.58
Hypertension	No	1,224	33.92
	Yes	2,385	66.08
Dyslipidemia	No	1,766	48.93
	Yes	1,843	51.07
Cancer	No	3,480	96.43
	Yes	129	3.57
Pulmonary disease	No	2,944	81.57
	Yes	665	18.43
Liver disease	No	3,271	90.63
	Yes	338	9.37
Heart disease	No	2,260	62.62
	Yes	1,349	37.38
Stroke	No	3,119	86.42
	Yes	490	13.58
Kidney disease	No	2,989	82.82
	Yes	620	17.18
Asthma	No	3,291	91.19
	Yes	318	8.81

### Univariate analysis

Table 3 compares depressive and non-depressive cases across various demographic, behavioral, and health-related indicators. The table reports the sample size and percentage for each subgroup under the non-depressive and depressive categories, along with chi-square test results to evaluate statistical significance. Age Group: The majority of participants in both depressive and non-depressive groups were aged 61–80 years. The chi-square test for age yielded a value of 4.048 with a corresponding *p*-value of 0.044, indicating a statistically significant difference in age distribution between the depressive and non-depressive groups at the 5% significance level. Cancer: In both depressive and non-depressive groups, the majority of participants did not have cancer. The chi-square test for cancer yielded a value of 2.244 with a p-value of 0.134, suggesting no significant difference in cancer status between the two groups. These findings indicate that while age is significantly associated with depressive symptoms, cancer status does not appear to have a significant impact. The chi-square tests provide a robust statistical basis for identifying variables potentially associated with depressive symptoms in older adults diabetic patients.

**Table 3:** Univariate analysis.

Category	Subcategory	Non-depressive symptoms (*n* = 2,214)		Depressive symptoms (*n* = 1,395)		*X* ^2^	*P*-values
		Sample size	Proportion (%)	Sample size	Proportion (%)		
Age	61–80 years	2,094	94.58	1,340	96.06	4.048	0.044
	≥81 years	120	5.42	55	3.94		
Education level	Primary school and below	1,423	64.27	1,117	80.07	110.545	*P* < 0.001
	Middle school	423	19.11	179	12.83		
	High school and above	368	16.62	99	7.1		
Gender	Female	1,097	49.55	924	66.24	96.725	*P* < 0.001
	Male	1,117	50.45	471	33.76		
Marital Status	Divorced/Widowed/Separated/Single	416	18.79	353	25.3	21.664	*P* < 0.001
	Married	1,798	81.21	1,042	74.7		
Region	Rural areas	954	43.09	855	61.29	113.400	*P* < 0.001
	Urban areas	1,260	56.91	540	38.71		
Residence area	Eastern region	918	41.46	419	30.04	53.019	*P* < 0.001
	Central region	819	36.99	572	41		
	Western region	477	21.54	404	28.96		
Smoking	No	1,224	55.28	923	66.16	42.038	*P* < 0.001
	Yes	990	44.72	472	33.84		
Drinking	No	1,550	70.01	1,091	78.21	29.309	*P* < 0.001
	Yes	664	29.99	304	21.79		
Sleep duration	Short sleep duration	537	24.25	620	44.44	161.070	*P* < 0.001
	Normal sleep duration	1,084	48.96	517	37.06		
	Long sleep duration	593	26.78	258	18.49		
Hypertension	No	796	35.95	428	30.68	10.612	0.001
	Yes	1,418	64.05	967	69.32		
Dyslipidemia	No	1,113	50.27	653	46.81	4.102	0.043
	Yes	1,101	49.73	742	53.19		
Cancer	No	2,143	96.79	1,337	95.84	2.244	0.134
	Yes	71	3.21	58	4.16		
Pulmonary disease	No	1,887	85.23	1,057	75.77	50.949	*P* < 0.001
	Yes	327	14.77	338	24.23		
Liver disease	No	2,044	92.32	1,227	87.96	19.205	*P* < 0.001
	Yes	170	7.68	168	12.04		
Heart disease	No	1,456	65.76	804	57.63	24.159	*P* < 0.001
	Yes	758	34.24	591	42.37		
Stroke	No	1,964	88.71	1,155	82.8	25.496	*P* < 0.001
	Yes	250	11.29	240	17.2		
Kidney disease	No	1,898	85.73	1,091	78.21	34.007	*P* < 0.001
	Yes	316	14.27	304	21.79		
Asthma	No	2,067	93.36	1,224	87.74	33.622	*P* < 0.001
	Yes	147	6.64	171	12.26		

*χ^2^* represents the Chi-square test. *P*-values < 0.05 are considered statistically significant.

### Baseline regression analysis

Table 4 reveals several significant factors influencing the outcome. Age ≥81 years shows a lower odds ratio (OR = 0.821) compared to those aged 61–8 0, though the result is not statistically significant (*p* = 0.275). Higher education levels, such as middle school (OR = 0.701, *p* = 0.001) and high school or above (OR = 0.561, *p* < 0.001), are associated with significantly lower odds of the outcome, indicating a protective effect. Gender differences also emerge, with males having lower odds of the outcome (OR = 0.589, *p* < 0.001). Marital status is marginally significant, as married individuals show a slightly lower odds ratio (OR = 0.837, *p* = 0.052). Region-specific differences are notable, with urban areas exhibiting significantly lower odds (OR = 0.551, *p* < 0.001), while central (OR = 1.451, *p* < 0.001) and western regions (OR = 1.653, *p* < 0.001) demonstrate higher odds of the outcome. Smoking, drinking, and certain chronic health conditions such as hypertension, dyslipidemia, cancer, and asthma do not show significant associations with the outcome, as their *p*-values exceed 0.05. In contrast, sleep duration, with both normal (OR = 0.535, *p* < 0.001) and long sleep durations (OR = 0.465, *p* < 0.001), is a significant protective factor. Among the health conditions, pulmonary disease (OR = 1.378, *p* = 0.002), stroke (OR = 1.446, *p* = 0.001), and kidney disease (OR = 1.479, *p* < 0.001) are significantly associated with higher odds of the outcome.

**Table 4:** Baseline regression results.

Category	Subcategory	OR	Std	*Z*	*P*-values	95%CI
Age	61–80 years	1	–	–	–	–
	≥81 years	0.821	0.148	−1.090	0.275	0.576–1.170
Education level	Primary school and below	1	–	–	–	–
	Middle school	0.701	0.075	−3.320	0.001	0.568–0.865
	High school and above	0.561	0.074	−4.410	*P* < 0.001	0.434–0.725
Gender	Female	1	–	–	–	–
	Male	0.589	0.069	−4.540	*P* < 0.001	0.468–0.740
Marital Status	Divorced/Widowed/Separated/Single	1	–	–	–	–
	Married	0.837	0.076	−1.940	0.052	0.700–1.001
Region	Rural areas	1	–	–	–	–
	Urban areas	0.551	0.043	−7.720	*P* < 0.001	0.473–0.641
Residence area	Eastern region	1	–	–	–	–
	Central region	1.451	0.125	4.310	*P* < 0.001	1.225–1.718
	Western region	1.653	0.160	5.180	*P* < 0.001	1.366–1.999
Smoking	No	1	–	–	–	–
	Yes	1.026	0.114	0.230	0.819	0.825–1.275
Drinking	No	1	–	–	–	–
	Yes	0.960	0.089	−0.440	0.659	0.801–1.151
Sleep duration	Short sleep duration	1	–	–	–	–
	Normal sleep duration	0.535	0.045	−7.410	*P* < 0.001	0.453–0.631
	Long sleep duration	0.465	0.047	−7.590	*P* < 0.001	0.381–0.566
Hypertension	No	1	–	–	–	–
	Yes	1.056	0.088	0.650	0.514	0.897–1.242
Dyslipidemia	No	1	–	–	–	–
	Yes	1.080	0.086	0.980	0.329	0.925–1.262
Cancer	No	1	–	–	–	–
	Yes	1.113	0.215	0.550	0.58	0.762–1.625
Pulmonary disease	No	1	–	–	–	–
	Yes	1.378	0.142	3.110	0.002	1.126–1.686
Liver disease	No	1	–	–	–	–
	Yes	1.281	0.163	1.940	0.052	0.998–1.645
Heart disease	No	1	–	–	–	–
	Yes	1.143	0.092	1.670	0.095	0.977–1.339
Stroke	No	1	–	–	–	–
	Yes	1.446	0.156	3.420	0.001	1.171–1.787
Kidney disease	No	1	–	–	–	–
	Yes	1.479	0.146	3.960	*P* < 0.001	1.219–1.795
Asthma	No	1	–	–	–	–
	Yes	1.257	0.176	1.640	0.102	0.9563–1.652

OR is the odds ratio, indicating the odds of the outcome occurring for each category relative to the reference category. Std is the standard error of the coefficient. *Z* is the *Z*-score, a statistical measure that represents the number of standard deviations an observation is from the mean. *P* is the *p*-value for the hypothesis test, indicating the statistical significance of each variable, with values less than 0.05 considered statistically significant. 95% CI is the 95% confidence interval.

### The regression results comparing three different years

Regarding the potential impact of the COVID-19 pandemic on the study results, we analyzed data from the years 2015, 2018, and 2020, with particular focus on the effect of the pandemic period (2020) on depressive symptoms in older adult diabetic patients in China. It is noteworthy that although depressive symptoms increased during the pandemic, the results from 2020 did not show significant deviations in the identified risk factors compared to 2015 and 2018. However, we observed a more pronounced prevalence of depressive symptoms in the 2020 cohort, especially among patients with comorbidities and those with shorter sleep durations. A comparison of results from all 3 years is provided in Supplementary Table 1 to further highlight the observed trends. These findings suggest that while the pandemic exacerbated the burden of depressive symptoms in older diabetic patients, the identified risk factors remained consistent across the years.

### Sensitivity analysis

Table 5 presents the results of the sensitivity analysis, which includes regression models using CES-D thresholds of 12 and 8 for classifying depressive symptoms. The table reports odds OR, standard errors, and *p*-values for both models. The findings show that the OR across different categories remain consistent with the baseline regression results, regardless of the CES-D threshold applied. This consistency demonstrates the robustness, reliability, and effectiveness of the model.

**Table 5:** Sensitivity analysis regression results.

Category	Subcategory	CES-D score with a threshold of 12			CES-D score with a threshold of 8		
		OR	Std	*P*	OR	Std	*P*
Age	61–80 years	1	–	–	1	–	–
	≥81 years	0.631	0.129	0.025	0.928	0.158	0.659
Education level	Primary school and below	1	–	–	1	–	–
	Middle school	0.732	0.085	0.007	0.702	0.071	<0.001
	High school and above	0.615	0.089	0.001	0.540	0.064	<0.001
Gender	Female	1	–	–	1	–	–
	Male	0.475	0.060	<0.001	0.601	0.067	<0.001
Marital Status	Divorced/Widowed/Separated/Single	1	–	–	1	–	–
	Married	0.817	0.077	0.032	0.871	0.079	0.126
Region	Rural areas	1	–	–	1	–	–
	Urban areas	0.529	0.043	<0.001	0.606	0.045	<0.001
Residence area	Eastern region	1	–	–	1	–	–
	Central region	1.466	0.135	<0.001	1.338	0.111	<0.001
	Western region	1.776	0.182	<0.001	1.618	0.153	<0.001
Smoking	No	1	–	–	1	–	–
	Yes	1.175	0.140	0.177	1.011	0.108	0.919
Drinking	No	1	–	–	1	–	–
	Yes	0.964	0.096	0.715	1.114	0.098	0.222
Sleep duration	Short sleep duration	1	–	–	1	–	–
	Normal sleep duration	0.523	0.046	<0.001	0.530	0.044	<0.001
	Long sleep duration	0.432	0.046	<0.001	0.455	0.045	<0.001
Hypertension	No	1	–	–	1	–	–
	Yes	1.066	0.094	0.466	1.111	0.089	0.187
Dyslipidemia	No	1	–	–	1	–	–
	Yes	1.035	0.087	0.683	1.064	0.082	0.417
Cancer	No	1	–	–	1	–	–
	Yes	1.1016	0.221	0.63	1.266	0.245	0.223
Pulmonary disease	No	1	–	–	1	–	–
	Yes	1.275	0.136	0.023	1.534	0.159	<0.001
Liver disease	No	1	–	–	1	–	–
	Yes	1.304	0.171	0.043	1.153	0.147	0.263
Heart disease	No	1	–	–	1	–	–
	Yes	1.202	0.101	0.029	1.113	0.087	0.174
Stroke	No	1	–	–	1	–	–
	Yes	1.498	0.166	<0.001	1.423	0.153	0.001
Kidney disease	No	1	–	–	1	–	–
	Yes	1.350	0.139	0.003	1.403	0.138	0.001
Asthma	No	1	–	–	1	–	–
	Yes	1.362	0.193	0.029	1.196	0.171	0.209

OR is the odds ratio, indicating the odds of the outcome occurring for each category relative to the reference category. Std is the standard error of the coefficient. *P* is the *p*-value for the hypothesis test, indicating the statistical significance of each variable, with values less than 0.05 considered statistically significant.

## Discussion

The findings of this study provide novel insights into the existing literature, particularly in the context of older adults in China. By examining the prevalence of depressive symptoms and its associated factors among elderly diabetic patients, this research highlights significant risk factors such as lower education levels, female gender, shorter sleep duration, and the presence of multiple comorbidities, which substantially increase the likelihood of depressive symptoms in this population. These results address a gap in the literature on depressive symptoms among older diabetic individuals in China, offering unique contributions within the socio-cultural and healthcare context of the country. The findings provide essential empirical data that can inform the development of targeted mental health interventions, particularly for high-risk groups within the aging population, thereby advancing public health strategies in China.

The study found that male older adult diabetic patients had a significantly lower risk of developing depressive symptoms compared to females. This finding is consistent with previous studies, which suggest that women are more susceptible to depressive symptoms due to physiological and psychological factors (Dye et al., 1995; Liu et al., 2024; Kuehner, 2017). AlOzairi et al. (2024) research indicates that female sex and microvascular complications are associated with an increased likelihood of depressive symptoms. This disparity is primarily driven by biological factors (e.g., hormonal fluctuations) and psychosocial influences (e.g., disparities in coping resources and exposure to social stress). Furthermore, with increasing gender role equality (e.g., expanded employment opportunities for women), gender differences in certain mental disorders have diminished in recent years; however, the gender disparity in depressive symptoms remains pronounced (Roy and Lloyd, 2012; Salk et al., 2017).

Regarding education level, the study consistently found that lower education levels were associated with a higher prevalence of depressive symptoms. Individuals with higher education levels (high school or above) exhibited a significantly lower risk of depressive symptoms compared to those with elementary school education or below. Lorant et al. (2003) suggested that lower education levels may increase the risk of depressive symptoms, potentially due to limited socioeconomic resources and inadequate coping strategies. Conversely, higher education levels may mitigate the risk of depression by enhancing health literacy and socioeconomic status (Moussavi et al., 2007). However, Bjelland et al. (2008) did not observe a significant association between education level and depressive symptoms, attributing the discrepancy to differences in study populations and social welfare systems.

Meng et al. (2012) demonstrated a significant association between depressive symptoms, hypertension, and dyslipidemia. These conditions may collectively contribute to cardiovascular dysfunction through mechanisms such as neuroendocrine dysregulation, autonomic nervous system dysfunction, inflammation, and oxidative stress, ultimately increasing the risk of cardiovascular disease (Meng et al., 2012; Paris et al., 2024; Jiang et al., 2020).

Additionally, studies by Fang et al. and Riemann et al. identified a bidirectional relationship between depressive symptoms and sleep disorders. Sleep disturbances, such as insomnia, are not only symptoms of depression but may also act as independent risk factors for its development. This relationship is likely mediated by mechanisms including inflammatory responses, circadian rhythm disruptions, and neurotransmitter imbalances, providing critical intervention targets for the prevention and treatment of depression (Fang et al., 2019; Riemann et al., 2020). While the study found that sleep duration and efficiency were not significantly associated with depressive symptoms, recent literature suggests that objectively measured sleep duration and sleep efficiency may not always correlate with depressive symptoms (Al-Ozairi et al., 2024). This discrepancy could arise from the differences in how sleep is measured (subjectively vs. objectively) and the complexities of depression’s relationship with sleep, which may not be directly influenced by sleep duration alone.

Chronic diseases such as stroke, kidney disease, and pulmonary disease have been identified as independent risk factors for depressive symptoms, consistent with the findings of Moussavi et al. (2007), who highlighted a strong association between multimorbidity and depressive symptoms. The negative impact of chronic diseases on patients’ quality of life, social participation, and mental health serves as a potential mechanism underlying their role as risk factors for depressive symptoms. Further analysis revealed that the effects of different chronic diseases on depressive symptoms vary. For instance, stroke patients exhibited a significantly higher prevalence of depressive symptoms compared to those with other chronic conditions. Using NHANES data and bioinformatics analysis, Yang explored the bidirectional relationship between depressive symptoms and stroke, identifying shared genetic mechanisms that suggest these conditions may influence each other through common genetic and molecular pathways, thereby uncovering the genetic basis of post-stroke depressive symptoms (Yang et al., 2024). Studies by Colita et al. (2024) and Xiao et al. (2024) further elucidated the molecular mechanisms of post-stroke depressive symptoms, including neuroinflammation, innate immune-mediated neuroinflammatory responses, and shared molecular pathways with depressive symptoms, emphasizing the central role of inflammation and immunity in the onset and progression of post-stroke depressive symptoms. Hackett et al. (2005) reported that the prevalence of depressive symptoms following a stroke ranges from 30 to 50%, which aligns with the findings of this study.

This study has several limitations. First, although the CHARLS database is nationally representative, it is subject to recall bias and inaccuracies in self-reported data, particularly for sensitive variables like depressive symptoms and chronic conditions. The duration of depressive symptoms, which could be influenced by comorbid conditions such as diabetes, was not assessed, limiting our ability to isolate the specific impact of diabetes. The cross-sectional design also prevents us from establishing causal relationships. Second, the exclusion of individuals with severe mental illness and missing data may have introduced selection bias, potentially underestimating the true prevalence of depressive symptoms. The dataset does not provide information on the timing of diabetes diagnosis, limiting our ability to assess its duration and impact on depressive symptoms. Third, while we accounted for many covariates, unmeasured variables such as genetic predisposition, life events, and access to mental health services could have influenced the results. Additionally, there may be concerns about the accuracy of depressive symptom reporting, especially among poorly educated women in rural areas. Although trained interviewers conducted the survey, misunderstanding of medical terms remains a limitation. Finally, we did not differentiate between type 1 and type 2 diabetes, which is a crucial limitation. These two types of diabetes have significant differences in their pathophysiology, management, and psychological implications. As the CHARLS dataset does not specify the type of diabetes for each participant, this omission limits the interpretability of our findings. Future studies should consider distinguishing between type 1 and type 2 diabetes to better understand their differential impacts on depressive symptoms and other related factors.

## Conclusion

This study highlights that certain subgroups of older adult diabetic patients, including women, individuals with low educational levels, those who are unmarried or widowed, residents of rural areas, and those with multiple chronic conditions, are at a higher risk for depression. These findings suggest that public health interventions and mental health screening strategies in China should prioritize these high-risk groups. Tailored mental health support and comprehensive care plans should be integrated into diabetes management for these populations, with particular attention to addressing their unique psychological and social challenges. Such interventions could improve both mental and physical health outcomes, ultimately enhancing the quality of life for older adults with diabetes.

## Data Availability

The data used in this study are publicly released data by CHARLS. Permissions were obtained to access the data used in our research, which were granted by the CHARLS team. The raw data is available on the website (https://charls.pku.edu.cn/en).

## Footnotes

^1^ http://charls.pku.edu.cn/index.htm
