# Supplementary material for: Prevalence and associated factors of depressive symptoms among older adult diabetic patients in China: a nationally representative cross-sectional study
Source: Front Psychol. 2025 Jun 27;16:1581603. doi: 10.3389/fpsyg.2025.1581603 (PMC12247175; doi:10.3389/fpsyg.2025.1581603)
Supplement: Supplementary file 1 [file Data_Sheet_1.pdf]

Supplementary Table 1: The regression results comparing three different years

Category	Subcategory	2015		2018		2020	
		OR	P	OR	P	OR	P
Age	61–80 years	1	-	1	-	1	-
	≥81 years	0.490	0.102	0.809	0.431	1.048	0.879
Education level	Primary school and below	1	-	1	-	1	-
	Middle school	0.516	0.008	0.707	0.048	0.781	0.138
	High school and above	0.507	0.024	0.606	0.018	0.544	0.003
Gender	Female	1	-	1	-	1	-
	Male	0.914	0.723	0.425	<0.001	0.615	0.010
Marital Status	Divorced/Widowed/Separated/Single	1	-	1	-	1	-
	Married	0.996	0.984	0.754	0.055	0.862	0.293
Region	Rural areas	1	-	1	-	1	-
	Urban areas	0.524	<0.001	0.567	<0.001	0.542	<0.001
Residence area	Eastern region	1	-	1	-	1	-
	Central region	1.995	0.001	1.220	0.157	1.439	0.007
	Western region	1.734	0.015	1.845	<0.001	1.457	0.012
Smoking	No	1	-	1	-	1	-
	Yes	0.724	0.179	1.100	0.598	1.161	0.404
Drinking	No	1	-	1	-	1	-
	Yes	0.933	0.756	1.145	0.369	0.836	0.208
Sleep duration	Short sleep duration	1	-	1	-	1	-
	Normal sleep duration	0.610	0.011	0.549	<0.001	0.482	<0.001
	Long sleep duration	0.441	0.001	0.443	<0.001	0.502	<0.001
Hypertension	No	1	-	1	-	1	-

	Yes	1.180	0.368	1.016	0.908	1.022	0.869
Dyslipidemia	No	1	-	1	-	1	-
	Yes	0.811	0.277	1.233	0.105	1.108	0.403
Cancer	No	1	-	1	-	1	-
	Yes	0.646	0.476	1.290	0.398	1.134	0.658
Pulmonary disease	No	1	-	1	-	1	-
	Yes	1.275	0.314	1.580	0.005	1.229	0.207
Liver disease	No	1	-	1	-	1	-
	Yes	0.969	0.931	1.098	0.645	1.657	0.008
Heart disease	No	1	-	1	-	1	-
	Yes	1.117	0.580	1.153	0.270	1.146	0.265
stroke	No	1	-	1	-	1	-
	Yes	1.428	0.288	1.433	0.029	1.495	0.013
Kidney disease	No	1	-	1	-	1	-
	Yes	3.078	<0.001	1.310	0.081	1.312	0.075
asthma	No	1	-	1	-	1	-
	Yes	1.192	0.614	1.306	0.224	1.222	0.356

OR is the odds ratio, indicating the odds of the outcome occurring for each category relative to the reference category. P is the p-value for the hypothesis test, indicating the statistical significance of each variable, with values less than 0.05 considered statistically significant.
